# Gender and Disparity in First Authorship in Cardiology Randomized Clinical Trials

**DOI:** 10.1001/jamanetworkopen.2021.1043

**Published:** 2021-03-09

**Authors:** Roxana Mehran, Ashish Kumar, Agam Bansal, Mariam Shariff, Martha Gulati, Ankur Kalra

**Affiliations:** 1 The Zena and Michael A. Wiener Cardiovascular Institute, Icahn School of Medicine at Mount Sinai, New York, New York; 2 Associate Editor, *JAMA Cardiology*; 3 Department of Critical Care Medicine, St. John’s Medical College Hospital, Bengaluru, India; 4 Department of Internal Medicine, Cleveland Clinic, Cleveland, Ohio; 5 Department of Cardiology, University of Arizona-Phoenix, Phoenix; 6 Department of Cardiovascular Medicine, Heart, Vascular and Thoracic Institute, Cleveland Clinic, Cleveland, Ohio; 7 Section of Cardiovascular Research, Heart, Vascular and Thoracic Department, Cleveland Clinic Akron General, Akron, Ohio

## Abstract

This cross-sectional study assesses the annual proportions and overall trend of female first authors in cardiology randomized clinical trials from 2011 to 2020.

## Introduction

The current dialogue in cardiovascular medicine is evolving to confer women with equal representation. According to the American College of Cardiology, women represent only 12% of the workforce in cardiology.^1^ Furthermore, only 9% of the interventional cardiology workforce are women. These numbers extrapolate into academia as well. Although a recent study reported a significant increase in the trend toward female first and senior authors in the cardiology peer-reviewed literature over the past 2 decades, the percentage has remained markedly low.^2^ This disparity was further substantiated by another study^3^ that highlighted the underrepresentation of women as first authors in heart failure randomized clinical trials (RCTs). The current literature lacks evidence on female first authors in cardiology RCTs over the past several years. The present study evaluated the annual proportions and overall trend of female first authors in cardiology RCTs from 2011 to 2020.

## Methods

In this cross-sectional study, we performed a PubMed search using the keyword *cardiology* and RCT as the article type from January 1, 2011, to October 18, 2020. The study was deemed exempt from review by the institutional review board at Cleveland Clinic. There was waiver of informed consent per institutional policy. The present study followed the Strengthening the Reporting of Observational Studies in Epidemiology (STROBE) reporting guideline.

The PubMed search was performed using the easyPubMed package in R, version 4.0.3 (R Project for Statistical Computing), which is an R interface for Entrez Programming Utilities aimed at facilitating programmatic access to PubMed. Gender of the first authors was assessed using the gender package in R, which encodes gender based on name and date of birth using the US Social Security Administration infant name data. The infant name data from 1932 to 2012 were used to encode gender based on first names in the present analysis. The proportions of female first authors are presented as percentages stratified by year. A Jonckheere trend test was used to assess the trend in the proportion of female first authors over the years.

## Results

With use of the easyPubMed package in R, the search identified a total of 8613 RCTs indexed in PubMed from 2011 to 2020: 930 RCTs in 2020, 1162 RCTs in 2019, 977 RCTs in 2018, 1235 RCTs in 2017, 1154 RCTs in 2016, 1022 RCTs in 2015, 776 RCTs in 2014, 518 RCTs in 2013, 504 RCTs in 2012, and 335 RCTs in 2011. Among the 8613 identified RCTs, the gender package was able to encode gender for 6189 (72%) first authors (gender was unidentifiable for 2424 first authors [28%]): 685 (74%) in 2020, 865 (74%) in 2019, 713 (73%) in 2018, 887 (72%) in 2017, 793 (69%) in 2016, 720 (70%) in 2015, 560 (72%) in 2014, 356 (69%) in 2013, 360 (71%) in 2012, and 250 (75%) in 2011. Of the 6189 RCTs, 1838 (30%) had women as first authors. Among the 1838 female first authors, 795 (43%) had distinctive first names, and among 4351 male first authors, 1167 (27%) had distinctive first names. In 2020, 238 of 685 RCTs (35%) had women as first authors; in 2019, 259 of 865 (30%); in 2018, 237 of 713 (33%); in 2017, 258 of 887 (29%); in 2016, 252 of 793 (32%); in 2015, 199 of 720 (28%); in 2014, 159 of 560 (28%); in 2013, 90 of 356 (25%); in 2012, 91 of 360 (25%); and in 2011, 55 of 250 (22%) (Figure). The Jonckheere trend test reported an increasing trend in the proportions of female first authors from 2011 to 2020.

**Figure.**
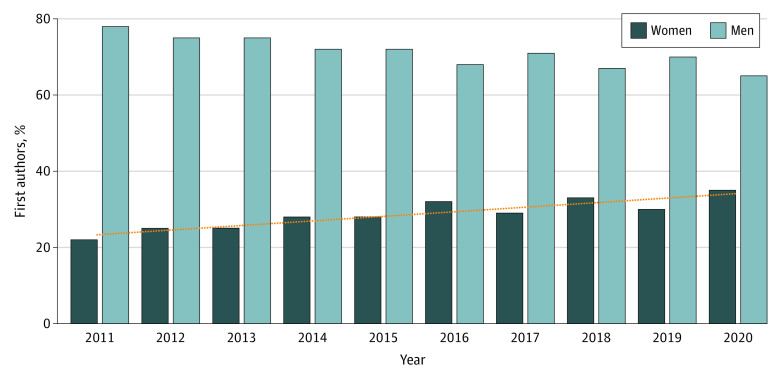
Women and Men First Authors in Cardiology Randomized Clinical Trials

## Discussion

The present study found that 30% women were first authors in cardiology RCTs from 2011 to 2020; this increase may be associated with advocacy for female empowerment and equal representation. Furthermore, considering the lower percentage of distinctive first author names among men, there is a possibility of a select few male authors first-authoring several RCTs. The higher percentage of distinctive first author names among women is indicative of greater diversity among female first authors. A major strength of this study was the methods used to identify the gender of the first author (ie, the gender package in R, which gave a probability of the gender of first authors). To our knowledge, previous studies^3^ did not delineate the methods used to identify the gender of authors. This study has limitations. First, only the PubMed database was searched for studies. Second, gender was encoded using the R package with US Social Security Administration infant name data from 1932 to 2020. First authors of studies from countries outside the US were assessed using these data, and this may have caused disparities. However, considering the multicultural ethnicity in the US, the use of US Social Security Administration infant name data appeared to be justifiable. Gender was unidentifiable for 28% of first authors using this database. However, these 2424 studies were distributed evenly over the years analyzed and thus are unlikely to have had an effect on the reported trend.

The number of women directing RCTs in cardiology reflects the scarcity of women in cardiology overall. A concerted effort to increase the inclusion of women as leaders in RCTs and to promote the inclusion of women in cardiology is needed.
